# Targeting class I histone deacetylase 2 in *MYC* amplified group 3 medulloblastoma

**DOI:** 10.1186/s40478-015-0201-7

**Published:** 2015-04-03

**Authors:** Jonas Ecker, Ina Oehme, Ralph Mazitschek, Andrey Korshunov, Marcel Kool, Thomas Hielscher, Judit Kiss, Florian Selt, Carina Konrad, Marco Lodrini, Hedwig E Deubzer, Andreas von Deimling, Andreas E Kulozik, Stefan M Pfister, Olaf Witt, Till Milde

**Affiliations:** Clinical Cooperation Unit Pediatric Oncology (G340), German Cancer Research Center (DKFZ), Im Neuenheimer Feld 280, 69120 Heidelberg, Germany; Center for Systems Biology, Massachusetts General Hospital, Boston, MA 02114 USA; The Broad Institute of Harvard and MIT, Cambridge, Massachusetts 02142 USA; Department of Neuropathology, University Hospital Heidelberg, Im Neuenheimer Feld 224, 69120 Heidelberg, Germany; Clinical Cooperation Unit Neuropathology (G380), German Cancer Research Center (DKFZ), and German Cancer Consortium (DKTK), Heidelberg, Germany; Division of Pediatric Neurooncology (B062), German Cancer Research Center (DKFZ), Heidelberg, Germany, and German Cancer Consortium (DKTK), Heidelberg, Germany; German Cancer Consortium (DKTK), Heidelberg, Germany; Division of Biostatistics (C060), German Cancer Research Center (DKFZ), Heidelberg, Germany; Section of Pediatric Brain Tumors, Department of Pediatric Oncology, Hematology and Immunology, University Hospital Heidelberg, Im Neuenheimer Feld 430, 69120 Heidelberg, Germany; Department of Pediatric Hematology, Oncology and Bone Marrow Transplantation, Charité - University Medicine Berlin, CVK, CC17, Augustenburger Platz 1, 13353 Berlin, Germany

**Keywords:** Medulloblastoma, HDAC, HDAC inhibitor, HDAC2, MYC

## Abstract

**Introduction:**

Medulloblastoma (MB) is the most frequent malignant brain tumor in children. Four subgroups with distinct genetic, epigenetic and clinical characteristics have been identified. Survival remains particularly poor in patients with Group 3 tumors harbouring a *MYC* amplification. We herein explore the molecular mechanisms and translational implications of class I histone deacetylase (HDAC) inhibition in MYC driven MBs.

**Material and Methods:**

Expression of HDACs in primary MB subgroups was compared to normal brain tissue. A panel of MB cell lines, including Group 3 *MYC* amplified cell lines, were used as model systems. Cells were treated with HDAC inhibitors (HDACi) selectively targeting class I or IIa HDACs. Depletion of HDAC2 was performed. Intracellular HDAC activity, cellular viability, metabolic activity, caspase activity, cell cycle progression, RNA and protein expression were analyzed.

**Results:**

HDAC2 was found to be overexpressed in MB subgroups with poor prognosis (SHH, Group 3 and Group 4) compared to normal brain and the WNT subgroup. Inhibition of the enzymatic activity of the class I HDACs reduced metabolic activity, cell number, and viability in contrast to inhibition of class IIa HDACs. Increased sensitivity to HDACi was specifically observed in *MYC* amplified cells. Depletion of HDAC2 increased H4 acetylation and induced cell death. Simulation of clinical pharmacokinetics showed time-dependent on target activity that correlated with binding kinetics of HDACi compounds.

**Conclusions:**

We conclude that HDAC2 is a valid drug target in patients with *MYC* amplified MB. HDACi should cover HDAC2 in their inhibitory profile and timing and dosing regimen in clinical trials should take binding kinetics of compounds into consideration.

**Electronic supplementary material:**

The online version of this article (doi:10.1186/s40478-015-0201-7) contains supplementary material, which is available to authorized users.

## Introduction

Medulloblastomas (MB) are the most frequent malignant brain tumors in children, with approximately 60 and 300 newly diagnosed cases per year in Germany and the US, respectively [1]. Brain tumors account for 38% of cancer-related deaths, while leukemias account for 24% only despite having a much higher incidence [2]. Current treatment strategies for MB include aggressive surgery, cranio-spinal irradiation and adjuvant chemotherapy dependent on a risk stratification, which until very recently was solely based on clinical features such as histology and presence or absence of metastates at the time of primary diagnosis (e.g. HIT 2000 trial in Germany). However, it has recently been recognized that MB comprises four distinct molecular subgroups termed WNT, SHH, Group 3 and Group 4 [3], and newly opened clinical trials, such as the SIOP PNET5 trial (NCT02066220), include molecular markers such as beta-catenin in their risk stratification. Both WNT and SHH groups have been classified based on their characteristic activated oncogenic pathways, yet much less is known about the drivers of Group 3 and 4 MB [4,5]. Importantly, the majority of Group 3 tumors are characterized by high protein levels of cMYC, either induced by *MYC* amplification or by aberrant *MYC* expression [6,7], and *MYC* amplification is a marker for high-risk in Group 3 [8].

Each molecular subgroup can be divided further into different subtypes based on characteristic molecular aberrations, with different clinical courses in SHH, Group 3 and Group 4 [8], strongly suggesting additional biological heterogeneity in each subgroup. Indeed, the analysis of molecular biomarkers in individual subgroups reveals complex heterogeneity of MB subgroups down to the individual level, as has been shown for SHH [9] and Group 3 [10] MB. While the WNT and the SHH subgroups are characterized by several recurrent focal mutations in their respective determining pathways, recurrent mutations are unexpectedly rare in Group 3 and Group 4 tumors [4,11,12]. However, several mechanisms of structural variation are recurrent in Group 3 and Group 4 tumors, including somatic copy number alterations, chromothripsis and tetraploidy [13,14], as well as a newly recognized mechanism termed enhancer hijacking that leads to aberrant oncogene expression [10].

More recently it has become evident that a driving element in Group 3 and Group 4 MBs are aberrations of genes associated with chromatin modification [5,15]. Most of these genes encode for histone mark reader proteins or members of chromatin modifying enzyme complexes, such as *KDM6A* [4], *MLL2* [11], *ZMYM3* and *CHD7* [12]. Somatic mutations, as well as aberrant expression and somatic copy number variations of chromatin modulators lead to altered H3K4 and H3K27 methylation profiles in Group 3 and Group 4 tumors [16]. Finally, the novel MB candidate driver gene *LIN28B* was identified in Group 3 and 4 MB solely based on aberrant DNA methylation and overexpression of an alternative transcript [17].

While much insight has been gained into the relevance and function of histone methylation-dependent epigenetic events in Group 3 and Group 4 MB, much less is known about lysine acetylation- (or HDAC-) dependent epigenetic aberrations in MB at a chromatin-wide level. The zinc-dependent HDAC1 through HDAC11 comprise 11 members grouped into four classes (I, IIa, IIb, and IV) [18]. In SHH MBs, SHH-induced HDAC activity is required for continued proliferation of cerebellar granule precursor cells [19]. We and others have previously shown that HDACi treatment exerts anti-tumoral effects in MB *in vitro* and *in vivo* [20-24]. Our group has shown that distinct HDAC family members control specific oncogenic functions in pediatric neuronal cancer models including differentiation, cell cycle regulation, apoptosis, autophagy, chemotherapy resistance [25,26], and alterations in tumor suppressor pathways [27,28].

We have further demonstrated that specific HDAC isoforms are differentially expressed in MB [29,30], and found that expression of class IIa HDACs 5 and 9 correlates with cytogenetic aberrations and poor clinical outcome in the entire cohort of MB tumors, and high HDAC2 expression in group 3 MBs [30]. With the recent advent of class-selective HDAC inhibitors (HDACis), such as the class IIa-selective HDACis MAZ1863 and MAZ1866 [31] and selective substrates has opened the possibility of class-selective exploration of HDAC biology.

The aim of the presented study is to investigate the selective targeting of HDAC family members in a MB subgroup specific manner, and to elucidate the translational consequences.

## Materials and methods

### Patients and clinical samples

Material from patients of tissue microarray (TMA) set (paraffin embedded medulloblastoma samples) were randomly collected at the Department of Neuropathology, Burdenko Neurosurgical Institute (Moscow, Russia) between 1993 and 2011. Approval to link laboratory data to clinical data was obtained by the Institutional Review Board. Two neuropathologists confirmed the diagnoses according to the 2000 WHO classification. None of the patients had received irradiation or chemotherapy before collection of specimens. Metastatic state (M stage) was determined by magnetic resonance imaging and cerebro- spinal fluid cytopathology at diagnosis. Clinical and histopathologic data are summarized in Additional file 1: Table S1.

### Cell lines, cell culture and siRNA-mediated knockdown

Cell lines and cell culture conditions have been described previously: MED8A, UW228-2, ONS76 and DAOY in [29], HD-MB03 in [24], D458 in [32]. All cell lines had their identity confirmed and proven to be free of contamination by mycoplasma or viral contamination using the Multiplex cell Contamination Test (McCT) service [33]. *MYC* status of all cell lines was confirmed by fluorescent in-situ hybridization (see below). siRNA transfection was performed as reported previously [29]. siRNA reagents were purchased from Qiagen (Hilden, Germany) (see Additional file 2: Table S2).

### RNA-isolation, cDNA synthesis, quantitative reverse transcription real-time PCR (qPCR) and gene expression analysis

RNA extraction, cDNA synthesis, quantitative real-time PCR, and software analysis was performed as reported previously [29]. Primers were purchased from Qiagen (see Additional file 3: Table S3). Normal cerebellum RNA was purchased from Clontech (Mountain View, CA, USA).

The database analysis tool R2 (http://r2.amc.nl) was used to investigate HDAC1, 2, and 3 mRNA expression in brain tumors and normal brain tissues using publicly available datasets (Dataset: [7], Probesets: HDAC1 (201209_at), HDAC2 (201833_at), HDAC3 (216326_s_at).

### Western blot (WB) and image processing

Protein concentrations of cell lysates were determined using the Thermo Scientific Pierce (Waltham, MA, USA) BCA Protein Assay Kit according to manufacturer’s instructions. The following antibodies were used: monoclonal mouse anti-human cMYC (1:200, catalog no. sc-40; Santa Cruz, Dallas, TX, USA), monoclonal mouse anti-human HDAC2 (1:1000; catalog no. sc-81599; Santa Cruz), polyclonal rabbit anti-human AcH4 (1:1000; catalog no. 06-866; Milipore, Billerica, MA, USA) and mouse monoclonal anti–β-actin (1:10000; catalog no. A5441; Sigma-Aldrich) and detected with Amersham ECL Prime Western Blotting Detection System (GE Healthcare, Little Chalfont, UK) on PVDF membrane with Chemi-Smart 5000 Technology (Vilber Lourmat, Eberhardzell, Germany). Uncropped images were contrast enhanced with Chemi-Capt 5000 (Vilber Lourmat) and subsequently cropped in Microsoft Office PowerPoint 2007 SP3 (Microsoft Corporation, Redmond, WA, USA).

### TMA, IHC, and fluorescent in-situ hybridization (FISH)

DNA and RNA was extracted from the original tumors included in this TMA and analyzed by nanoString [34] and/or 450 k Array [35] as described previously to assign the molecular subgroups. For preparation of the TMA and IHC, see [36]. For detection of HDAC2, the antibody No. ab32117 [Y461] (Abcam, Cambridge, UK) was used at 1:250 dilution. IHC was performed as reported previously [37]. The scoring of the IHC was performed by two investigators (JE and TM), who were both blinded to the clinical information. Four staining intensity levels were defined and weightened with 0 for no staining, 1 for weak staining, 2 for intermediate staining and 3 for strong staining. The H-Score was calculated by summation of the percentages of area stained at each intensity level multiplied by the weightened intensity (i.e. 0, 1, 2, or 3) [38]. The arithmetic mean of the scoring of both investigators was calculated.

*MYC* status of all cell lines was verified by FISH, as described previously [39]. The probe used was Vysis LSI MYC (Cat. No.: 03 N87-020, Abbott, Abbott Park, IL, USA).

### Microscopy

Bright-field images as well as IHC images were captured using an Olympus CX41 microscope with a Color View camera, and CellB 2.3 software (Olympus, Shinjuku, Tokyo, Japan).

### HDAC inhibitors (HDACis)

Class IIa HDACis MAZ1863 (compound 6) and MAZ1866 (compound 13) have been described previously [31]. MAZ1863 and MAZ1866, vorinostat (suberoylanilide hydroxamic acid, SAHA; Cat. No. S1047, Selleck Chemicals, Houston, TX, USA), and MS-275 (Cat. No. M4693-15A.25, Biomol GmbH, Hamburg, Germany) were dissolved in DMSO. HDACi and solvent controls were diluted in cell culture medium and added to the cell culture medium to achieve the indicated concentrations.

### HDAC activity assay and washout experiments

0.5×10*5 cells in 100 μl medium were seeded in a 96-well plate and incubated for 24 hours at 37°C, 5% CO_2_. The artificial class I HDAC substrate Boc-Lys(Ac)-AMC (Cat. No. 1875, Bachem, Bubendorf, Switzerland), or class IIa HDAC substrate Boc-Lys(trifluoroacetyl)-AMC (Cat. No. 1985, Bachem) reconstituted in DMSO were diluted in cell culture medium for a final concentration of 50 μM. The developer solution was prepared with a working concentration of 2.5 mg/ml trypsin from porcine pancreas in DMEM, 2% of TritonX-100 and 10 μM of Trichostatin-A (TSA) and stored on ice until usage. Cell culture media was carefully removed and cells were treated with varying concentrations of HDACi (25 μl/well). After 5 min incubation at room temperature 25 μl of substrate dilution were added. After 30 min incubation at 37°C 50 μl of developer solution were added. After 30 min incubation at 37°C emitted fluorescence was measured in the plate reader FLUOstar OPTIMA (BMG Labtech, Ortenberg, Germany), and analyzed with OPTIMA Software, Version 2.00 R3 (Labtech).

For washout experiments cells were seeded as described above. After 24 hours incubation at 37°C the medium was carefully removed and the cells were treated with a dilution of an HDAC inhibitor and incubated at 37°C. After 90 minutes the inhibitor was carefully removed from the cells and cells were washed three times with 200 μl of fresh medium. At timepoints 0 h, 1 h, 3 h, 6 h, 12 h and 24 h after inhibitor removal HDAC activity was measured as described above.

### Metabolic activity assay, cell counts and analysis of viability

The WST-1 assay (Roche, Basel, Switzerland) was used for the metabolic activity assays, these were performed as described [40]. Cell counts and analysis of viability by trypan blue exclusion staining were performed using a ViCell XR counter (Beckman Coulter, Brea, CA, USA).

### Measurement of the sub-G0 fraction and caspase-3–like activity

The sub-G0 fraction and caspase-3–like activity was measured as described [29]. The positive control for the caspase-3-like activity consisted of untransfected cells treated with UV light (35 mJ/cm^2^) 12 h before caspase-3-like activity measurement.

### Statistics analysis and graph editing

In vitro experiments were performed in a minimum of three biological replicates. Half-maximal effective concentrations (EC_50_) of HDACi were calculated using GraphPad Prism version 5.01 (GraphPad Software, La Jolla, CA, USA) for Windows. Results of treatments were compared using an unpaired t-test or One Way ANOVA test with Bonferroni’s multiple Comparison Test as indicated. p-values <0.05 were considered significant. Graphs were generated using GraphPad Prism version 5.01 and Microsoft Powerpoint for Mac 2011, Version 14.4.5.

## Results

### *MYC* amplified medulloblastomas display differential expression of class I HDACs

We have previously shown that differential expression of HDAC family members occur in medulloblastoma. Whereas class IIa HDACs 5 and 9 correlate with specific cytogenetic aberrations and poor clinical outcome in the entire cohort of MB tumors [29], analysis of MB subgroup specific expression revealed a particular high level of HDAC2 in all three subgroups of MB associated with higher risk, i.e. SHH, group 3 and group 4 [30].

**Figure 1:**
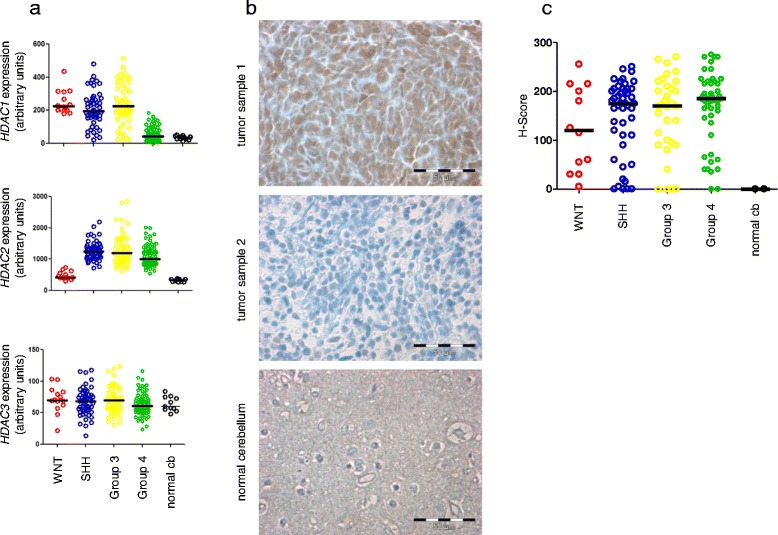
***HDAC2*** **mRNA and protein is differentially expressed in clinical medulloblastoma samples. a** scatter plot of mRNA expression of *HDAC1*, *2* and *3*, as measured by gene expression profiling. Only *HDAC2* is elevated in in the subgroups with poor prognosis (SHH, Group 3 and Group 4), but not in the subgroup with good prognosis (WNT) or normal cerebellum. **b** HDAC2 protein expression is detected by immunohistochemistry (IHC) (brown staining) in the majority of medulloblastoma tumor samples. Both positive (sample 1, molecular subgroup: group 3) and negative (sample 2, molecular subgroup: SHH) examples are shown. No HDAC2 protein expression is detected by IHC in normal cerebellum in the two samples analyzed (depicted region: molecular layer of the hemisphere). Scale bar size: 50 μm. **c** Semiquantitative analysis of HDAC2 IHC reveals higher expression of HDAC2 protein in SHH, Group3 and Group4 compared to WNT and normal cerebellum. cb: cerebellum.

Since target expression in the respective tissue is a pre-requisite for targeted treatment, we examined the expression of class I HDACs in primary tissues. In a first step, we investigated mRNA expression levels of class I HDACs *HDAC1*, *2* and *3* in primary MB samples. *HDAC2* was most abundantly expressed in MB (WNT: 296.2 – 742.4 AUs, SHH: 720.1 - 2190.1 AUs, Group3: 611.6 – 2847.6 AUs, Group4; 546.2 - 2007.1 AUs), compared to *HDAC1* (WNT: 179.3 – 436 AUs, SHH: 22.7 – 481.1 AUs, Group3: 22.2 – 514.3 AUs, Group4 4.4 – 184.9 AUs) and *HDAC3* (mean of WNT 21.6 – 103.3 AUs, SHH 13.7 – 118.1 AUs, Group3 30.9 – 123,5 AUs, Group4 23.9 – 116 AUs) (Figure 1a), with highest expression of *HDAC2* mRNA in subgroups with poor prognosis (SHH, Group 3 and 4), while both WNT and normal cerebellum showed low expression of *HDAC2* (Figure 1 a). In contrast, *HDAC1* mRNA displayed similar levels in subgroups with highly disparate prognoses (WNT and SHH/Group3), and at higher levels in WNT, SHH, and Group 3 than in Group 4 and normal cerebellum (Figure 1a, upper panel). Finally, *HDAC3* was expressed at relatively low but similar levels in all four molecular subgroups and normal cerebellum (Figure 1a, lower panel). *HDAC2* therefore displays the most differential expression pattern in the respective subgroups and is highly expressed in subgroups with overall poor clinical prognosis. In contrast both *HDAC1* and *3* expression shows little or no correlation to subgroup prognosis. We therefore focused on HDAC2 in the following analyses. To evaluate protein expression of the putative target HDAC2 in a large set of primary MB tumor samples, we performed immunohistochemistry (IHC) for HDAC2 on a tissue micro-array (TMA) with n = 142 MB samples (Figure 1b). Each sample was scored for HDAC2 staining using the H-Score in a blinded manner by 2 independent investigators. SHH, Group 3 and Group 4 MBs exhibited a higher H-Score compared to WNT and normal cerebellum (Figure 1c). In summary HDAC2 appears to be the most promising target within the tested class I HDACs, and HDAC2 protein is highly expressed in molecular groups with poor prognosis, including Group 3 which includes most of the *MYC* amplified cases.

**Figure 2:**
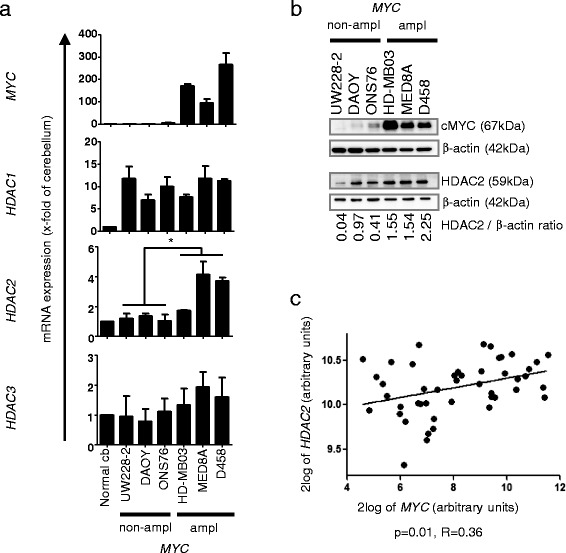
***MYC*** **amplified medulloblastoma cell lines show differential expression of class I HDACs HDAC1, 2 and 3. a** gene expression as measured by quantitative RT-real time PCR shows differential expression of *HDAC1*, *2* and *3* in medulloblastoma cell lines with high *MYC* expression, with HDAC2 showing a significant difference between *MYC* amplified and *MYC* non-amplified cell lines (* = p < 0.05, one way ANOVA test). **b** protein detection by western blot detects higher cMYC and HDAC2 protein in cells with *MYC* amplification. **c** correlation of *HDAC2* and *MYC* mRNA expression as measured by gene expression profiling in n = 42 primary Group 3 MB tumors (Pearson product-moment correlation coefficient, two tailed p-test). Bars depict mean and standard error of three independent experiments.

To investigate how well the MB cell lines used in our studies resemble the molecular biology of primary tumors we compared HDAC1, 2 and 3 and MYC gene and protein expression levels. Increased expression of both *MYC* mRNA and cMYC protein was confirmed in cell lines with *MYC* amplification by RT-qPCR and WB, respectively (Figure 2a, b). Similar to Group 3 MB tumors [30], the *MYC* amplified cell lines HD-MB03, MED8A, and D458 express elevated levels of class I HDACs 1,2,3 mRNA relative to normal cerebellum (Figure 2a). Additionally, only *HDAC2* showed a significant differential expression between *MYC* amplified compared to non-amplified cells (Figure 2a), which was confirmed on the protein level by western blot (Figure 2b). This correlation between *MYC* and *HDAC2* mRNA expression can also be found in primary Group 3 MBs in a series of n = 42 tumors (Figure 2c), GEO ID GSE37382 [14]. We conclude that the *MYC* amplified cell lines HD-MB03, MED8A, and D458 reflect the molecular biology of *MYC* amplified Group 3 MBs, with regard to expressing high levels of class I HDAC2, and thus are good models to study the function of HDAC2 in this subgroup.

### Inhibition of class I but not class IIa HDAC catalytic activity affects *MYC* amplified medulloblastoma cells

**Figure 3:**
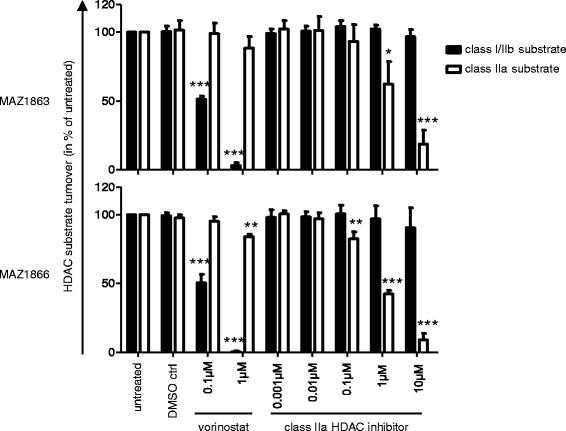
**HDAC inhibitors MAZ1863 and MAZ1866 are highly selective and potently inhibit class IIa activity.** Both MAZ1863 (top) and MAZ1866 (bottom) significantly inhibit intracellular class IIa HDAC activity compared to DMSO control, as measured by class IIa HDAC specific substrate turnover in a cell-based biochemical HDAC activity assay after treatment of MED8A MB cells with the compounds at indicated concentrations for 1 h. Class I/IIb HDAC activity is not inhibited by MAZ1863 or MAZ1866, while vorinostat significantly inhibits class I/IIb HDAC activity, as expected. Bars depict mean and standard error of three independent experiments, significant differences compared to DMSO control are indicated: * = p < 0.05, ** = p < 0.01, *** = p < 0.001 (unpaired t-test).

**Figure 4:**
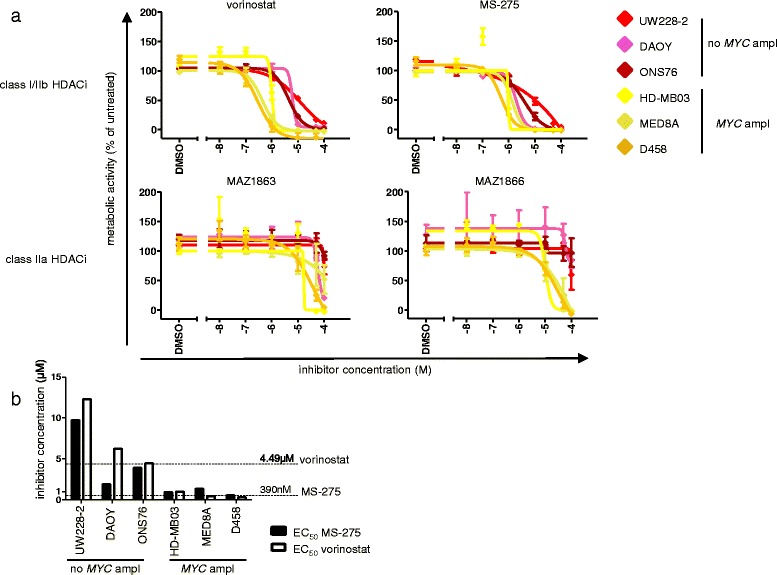
**Class I/IIb- but not class IIa-HDAC inhibitors potently inhibit metabolic activity of** ***MYC*** **amplified medulloblastoma (MB) cells in vitro. a** The metabolic activity of *MYC* amplified medullobastoma cells HD-MB03, MED8A, D458 (yellow) is potently inhibited by class I/IIb HDACi vorinostat and HDAC1,2,3 selective MS275, but not by class IIa HDACi MAZ1863 and MAZ1866, each after 72 h of treatment. The metabolic activity of MYC non-amplified MB cells UW228-2, DAOY, ONS76 (red) is inhibited only at very high concentrations. **b** EC_50_ values of vorinostat and MS-275 are significantly lower for *MYC* amplified cell lines, and are close to (MS-275) or below (vorinostat) published plasma concentrations clinically achievable in patients (dashed lines).

**Figure 5:**
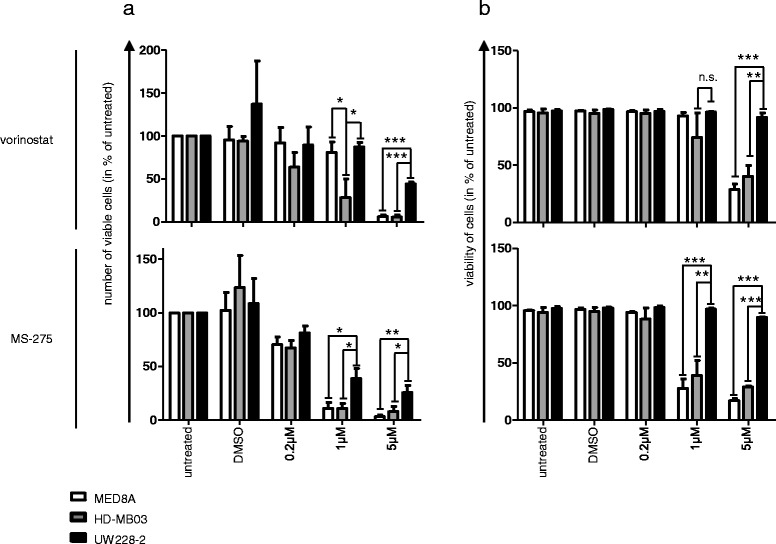
**Class I HDAC inhibitors potently inhibit cell growth of** ***MYC*** **amplified medulloblastoma (MB) cells in vitro.** Bars and error bars depict mean and standard error of numbers of viable cells **a** and cell viability (number of viable cells/number of total cells) **b** after 72 h of treatment, as determined by trypan blue staining. Class I selective HDAC inhibitors vorinostat (top) and MS-275 (bottom) potently inhibit cell growth of MYC amplified MB cells (MED8A and HD-MB03), while cell growth of MYC non amplified MB cells (UW228-2) is reduced only at higher concentrations. Bars depict mean and standard error of three independent experiments, significant differences between cell lines are indicated: * = p < 0.05, ** = p < 0.01, *** = p < 0.001 (unpaired t-test). ampl: amplified

To determine the functional oncogene dependency of MB cells to particular HDAC family members, we investigated the consequences of targeting HDAC enzymatic activity on MB cell survival in an isoform-selective manner. To this aim, we tested the ability of vorinostat (targeting class I and class IIb HDACs) [41] versus MAZ1863 and MAZ1866 (targeting class IIa HDACs) [31] to inhibit intracellular HDAC acitivity using class-specific substrates in a cell-based *in vitro* assay (Figure 3). As expected [41], vorinostat efficiently blocked class I/IIb enzymatic HDAC activity in MED8A MB cells (Figure 3, black bars), but not class IIa HDAC enzymatic activity (Figure 3, white bars). Vice versa, class IIa selective compounds MAZ1863 and MAZ1866 efficiently blocked intracellular class IIa HDAC enzymatic activity (Figure 3, white bars) but not classI/IIb HDAC activity (Figure 3, black bars). After having shown that MB cells harbor measurable intracellular class I, IIa, and IIb HDAC enzymatic activity that can selectively be blocked through small molecule inhibitors, we evaluated the anti-tumoral effect of HDACis with different HDAC isoform selectivity profiles (MAZ1863, MAZ1866, vorinostat and MS-275) on MB cells, we profiled MB cell lines with and without *MYC* amplification in a metabolic activity assay. After 72 h of treatment with HDACis, only very weak effects on metabolic activity could be observed for class IIa HDACis MAZ1863 and MAZ1866, both in *MYC* amplified and non-amplified MB cells (Figure 4a). In contrast, HDACis vorinostat (prefentially inhibiting class I/IIb HDACs) [41] or the class I specific inhibitor MS-275 (selctively inhibting HDAC1, 2, and 3) [41] greatly reduced metabolic activity in both *MYC* amplified and non-amplified MB cells (Figure 4a). However, comparison of the dose-dependent inhibition of metabolic activity by vorinostat and MS-275 for each cell line revealed a strong difference between *MYC* amplified and non-amplified MB cells, with EC_50_ values for *MYC* amplified cells within the range of published clinically achievable peak plasma concentrations (Figure 4b; Additional file 4: Table S4) (4,49 μM for vorinostat [42] and 390 nM for MS-275 [43]). Accordingly, after treatment for 72 h both the number as well as the viability of cells was significantly reduced in *MYC* amplified cells (MED8A, HD-MB03) compared to a *MYC* non-amplified cell line (UW228-2) (Figure 5). These results suggest that inhibition of class IIa activity has only a minor impact on MB tumor cell survival, whereas inhibition of class I enzymes HDAC1, 2 and 3 elicits a strong response in *MYC* amplified MB cells.

### HDAC2 depletion induces cell death and attenuates cell growth

**Figure 6:**
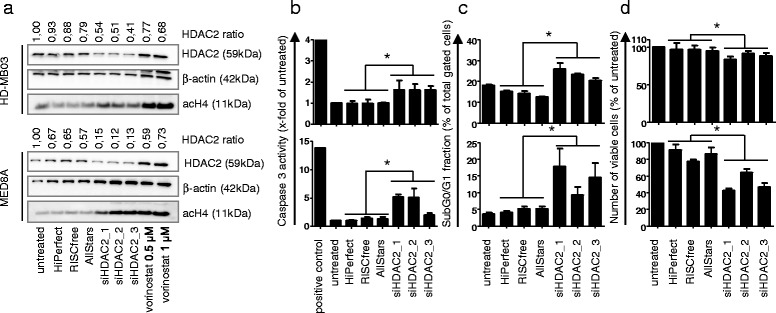
**siRNA-mediated knockdown of HDAC2 induces acetylation of histone 4, caspase 3-like activity and sub-G0/G1 fraction, and reduces cell number of** ***MYC*** **amplified MB cells. a** siRNA-mediated knockdown reduces HDAC2 protein and induces acetylation of histone 4 (H4) in *MYC* amplified cells MED8A and HD-MB03 72 h after transfection, as determined by western blot. HDAC2 ratio: HDAC2/beta-actin ratio, quantification of HDAC2 protein relative to untreated. Of note: The upper band in the beta-actin labeled blot of HD-MB03 cells is the signal from the HDAC2 detection, which was performed before the beta-actin detection. **b-d** siRNA-mediated knockdown induces caspase 3-like activity (72 h after transfection) and Sub-G0/G1 fraction (96 h after transfection), and reduces the number of viable cells (96 h after transfection) in *MYC* amplified cells MED8A and HD-MB03, as measured by caspase 3-like activity assay **b**, Nicoletti staining and flow cytometric analysis **c**, and trypan blue staining **d**, respectively. Bars depict mean and standard error of three independent experiments, significant differences are indicated: * = p < 0.05 (one way ANOVA test).

To investigate the effect of loss of HDAC2 function on tumor biology, we performed siRNA-mediated knockdowns of HDAC2 in *MYC* amplified cells. Knockdown with three different siRNAs against HDAC2 reduced HDAC2 protein, and increased acetylation of histone H4 72 h after knockdown (Figure 6a). Caspase 3-like activity was increased 72 h after knockdown (Figure 6b), both increasing the sub G0/G1 fraction (Figure 6c) and reducing the number of viable cells (Figure 6d) 96 h after knockdown. We therefore conclude that siRNA-mediated depletion of HDAC2 protein induces cell death and reduces cell growth.

### Simulation of clinical pharmacokinetics of HDACi in vitro uncovers challenges for translation

**Figure 7:**
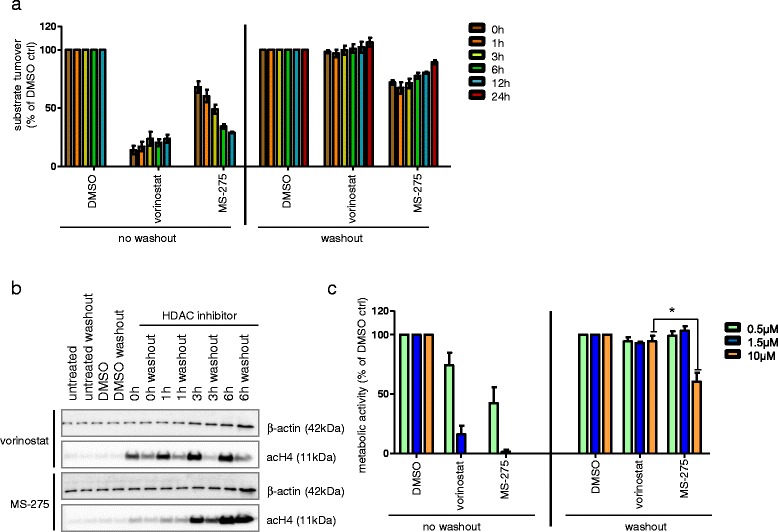
**In vitro simulation of clinical pharmacokinetics uncovers crucial hurdles of in vivo HDAC treatment. a** reduction of class I HDAC activity after in vitro treatment of MED8A cells with vorinostat or MS-275 measured at times indicated after washout of HDACi as determined by HDAC activity assay. HDAC activity is strongly reduced immediately upon vorinostat treatment, however immediately returns to normal after a 90 min incubation period followed by washout. HDAC activity is reduced in a time-dependent manner upon treatment with MS275, and returns to normal approx. 24 h after a 90 min incubation period followed by washout. **b** Incubation with vorinostat induces immediate and strong acetylation of histone 4 (acH4), which is quickly reduced after a 90 min incubation period followed by washout. Incubation with MS275 induces acetylation of histone 4 (H4) in a time dependent manner, which is stable even after a 90 min incubation period followed by washout. Actin served as loading control. **c** Incubation for 72 h with vorinostat or MS-275 induces strong reduction of metabolic activity, but this effect is nullified if metabolic activity is measured after 72 h with an incubation period of 90 min followed by washout once every 24 h. Bars depict mean and standard error of three independent experiments, significant differences are indicated: * = p < 0.05 (unpaired t-test).

Despite the well-established pre-clinical effects of HDAC inhibitors on differentiation, cell cycle, apoptosis, autophagy, chemotherapy resistance [25,26], as well as alterations of tumor suppressor pathways [27,28], clinicial trials have so far failed to demonstrate a significant anti-tumoral effect of HDACis in solid malignancies [44,48], in contrast to leukemias and lymphoma. One potential explanation concerns the very short *in vivo* half-life of some compounds including vorinostat with a plasma half-life of 90 minutes only [49], leaving the tumor unexposed to the compound for most of the time when dosed once per day. In classical cell culture models, however, cells are exposed to HDACi 24 h/day *in vitro*. To model the *in vivo* plasma half-life in our *in vitro* cell culture model, we performed wash-out experiments *in vitro*, simulating the *in vivo* conditions of a once per day vorinostat application. After incubation with either vorinostat or MS-275, intracellular class I/IIb HDAC activity was measured at regular intervals, starting immediately after the washout, and a setting with no washout served as controls. Vorinostat immediately and strongly suppressed HDAC activity in the setting with no washout (Figure 7a), demonstrating immediate inhibition of class I HDACs. MS-275 similarly suppressed HDAC activity only in a time-dependent manner, i.e. a level of suppression similar to vorinostat was only achieved after 12 h (Figure 7a), suggesting a slow inhibition kinetic of MS-275, as has already been shown for aminoanilide/benzamide based inhibitors [50]. In the washout setting, strikingly, the inhibition of HDAC activity by vorinostat was immediately lost after the washout, suggesting very fast binding kinetics and dissociation of the inhibitor from its target. MS-275 however did still inhibit HDAC activity after washout, similar to the level in the no washout setting at 0 h, with only a slow recovery of HDAC activity (Figure 7a), again suggesting slow binding kinetics. Accordingly, in the analysis of downstream epigenetic effects by WB of acetylated histone H4, we found an immediate and lasting hyperacetylation of H4 following vorinostat treatment in the no washout setting, which was rapidly reduced in the washout setting (Figure 7b). Conversely, the hyperacetylation of H4 in response to MS-275 treatment increased over time with almost no immediate hyperacetylation, but a long lasting effect even in the washout setting, i.e. hyperacetylation of H4 can still be found 6 h after washout (Figure 7b). Analysis of anti-tumoral efficacy as determined by metabolic activity after 72 h showed a strong effect in the no washout setting for both vorinostat and MS-275, as previously shown (Figure 7 c). However, if the cells were incubated once per 24 h for 90 min followed by a washout, simulating the clinical situation of once per day (qd) vorinostat application in a patient, no reduction of metabolic activity was seen for vorinostat, and only at a very high concentration of 10 μM for MS-275 (Figure 7c). In summary, vorinostat has a strong but short-lived inhibitory effect on HDAC activity, with a brief downstream effect on the epigenetic target H4 under simulated clinical conditions, which is reflected by no detectable effect on metabolic activity. MS-275 has a moderate but longer-lived effect on HDAC activity, with a lasting downstream effect on the epigenetic target H4 under simulated clinical conditions. This however translates into reduction of metabolic activity only at very high MS-275 concentrations, which are clinically not achievable. These findings suggest a clinically relevant correlation between a compound’s binding kinetics and its on-target activity.

## Discussion

Successful treatment of MB remains a challenge in many patients, which suffer from therapy-related side effects, and the prognosis remains poor for many patients with Group 3 MB [13]. Based on reports suggesting that epigenetic events seem to play an important role in this subgroup, we have investigated the selective targeting of HDAC family members in a subgroup specific manner.

We have previously found that the class IIa isoforms HDACs 5 and 9 are widely expressed on the protein level, and relatively overexpressed on the mRNA level in MB with poor prognosis (Chr 6q gain or 17q gain) [29]). Depletion of either HDAC5 or HDAC9 protein in MB cells resulted in a reduction of cell proliferation and increase in cell death [29]. To investigate whether inhibition of the enzymatic deacetylase activity can phenocopy these effects, we tested novel selective class IIa HDAC inhibitors. Although these inhibitors strongly inhibited class IIa enzymatic activity in MB cells in dose-dependent fashion, we did not observe any obvious biological effect, such as reduction of metabolic activity in MB cells. These results suggest that the inhibition of class IIa enzymatic activity clearly did not confer the same biological effect as the reduction of HDAC5 or HDAC9 protein, suggesting that the enzymatic activity is not the main mechanism for the oncogenic effects of HDAC5 or 9 in MB cells. In contrast, inhibition of class I HDACs elicited a strong response, especially in *MYC* amplified cell lines. The enzymatic activity of class I and IIa HDACs therefore plays very divergent roles in MB biology. Indeed, a tyrosine residue in the catalytic site of class I HDACs potentiates the lysine deacetylation activity acting as a transition stabilizer [51]. Due to a highly conserved mutation in the catalytic site of vertebrate class IIa HDACs, switching this tyrosine to a histidine residue, the deacetylating activity of class IIa HDACs on histone proteins is reduced more than a 1000-fold [52]. The repressive effect of class IIa HDACs on gene expression therefore appears to be largely independent of their catalytic activity on histone proteins. This surprising finding is further emphasized by studies showing that a splice variant of HDAC9 lacking the catalytic HDAC domain represses the expression of MEF2 target genes just as effectively as wild-type HDAC9 protein [53]. Finally it is being discussed that class IIa HDACs might play an important role in signal transduction independently of their enzymatic activity, either by bromodomain functioning as readers of epigenetic marks [41], or by shuttling between the nucleus and the cytoplasm [54] as has been shown for HDAC5 [55] and HDAC7 [56], and this could well be true for HDAC5 and 9 in MB.

To verify target presence and elucidate the class I HDACs involved in MB biology, we carried out expression analyses and found class I HDAC2 to be the most strongly overexpressed in MB in general [30] and in the three MB subgroups SHH, group 3 and group 4 (associated with unfavourable or high risk) in particular. Our results indicate that *MYC* amplified cell lines have higher sensitivity to HDACi, that comprise class I HDACs 1, 2 and 3 in their inhibitory profile, than *MYC* single copy cell lines. A previous report studying the effect of the HDACi depsipetide (FK228), which most potently inhibits all class I HDACs, demonstrated that the tumor most sensitive to depsipeptide treatment tested (a CNS-PNET) had the highest expression of *HDAC2* relative to *HDAC1*, and *3-7* [57]. Furthermore, *MYC* amplification is a hallmark of Group 3 MB, and importantly the transcription factor cMYC has been described to govern the transcription of *HDAC2* [58]. We have previously shown that the epigenetic regulation of miR-183 in neuroblastoma involves MYCN and HDAC2 in the same complex [27]. Based on these findings and consistent with our data, showing a significantly increased sensitivity for class I HDAC inhibiting agents in *MYC* amplified and HDAC2 overexpressing cell lines, the treatment of *MYC* amplified MB with HDAC inhibitors comprising class I HDACs in their inhibitory profile seems to be promising. Future studies should aim at the elucidation of the molecular interactions of cMYC and HDAC2, such as protein-protein interactions, feedback loops, and non-histone lysine deacetylation, governing the susceptibility of *MYC* amplified MB to HDACi.

Finally, whether HDACis will be efficacious for the treatment of solid tumors is still under debate. Many trials have failed to show meaningful response of solid tumors to HDACis [44] [45-48]. The root of failure to translate pre-clinical findings in general has been extensively discussed [59-61]. In general, insufficient pharmacological modeling of the clinical situation in terms of drugs concentrations and kinetics are the primarily criticized factors [61]. Detailed recommendations to improve the predictive value of pre-clinical cancer studies have been developed [60], which include the use of appropriate models, and understanding of the clinical reality, i.e. knowledge of the limitations of pre-clinical experimental settings. Analysis of the models used in our studies confirmed the faithful recapitulation of the patients’ tumors by the Group 3 cell lines used in our study, as evidenced by *MYC* amplification, and *MYC* and *HDAC2* expression analysis. To emulate *in vitro* the drug concentrations present in patients when treated with HDACis, we mimicked the clinical situation with appropriately low HDACi concentrations as well as washout experiments. Our results suggest that several reasons contribute to the failure of HDACis in the clinic despite their promising *in vitro* results. First, in the absence of a predictive biomarker, trial cohorts have been poorly pre-selected for patients responding with high sensitivity to HDACi treatment, suggesting the lack of efficacy. The most promising predictive biomarker identified in post-hoc analyses to date is IHC for HR23B, which has been shown to be a positive predictive marker for vorinostat in patients with cutaneous T-cell lymphoma [62] and for belinostat (PXD101) in patients with hepatocellular carcinoma [63]. We here validate *MYC* amplification as a predictive and routinely applicable clinical biomarker for HDACi sensitivity of medulloblastoma patients. Second, HDACis have a very short inhibitory effect on HDACs when present only a fraction of a day. As this is the case in patients, but not in cell culture, both pre- and clinical studies need to recognize clinical as opposed to cell culture conditions, and subsequently of the limits of pre-clinical data. Pre-clinical studies need to investigate conditions present in patients, as performed in our washout experiments, and especially pay attention to the lifetime of the drug-target complex [64], as well use adequate and informative readouts [65]. New dosing schedules could well be tested under these conditions, before being translated into the clinical setting. The difference of the half-life of the drug-target complex between vorinostat and MS-275 indicates that the lifetime of the drug-target complex is at least partially determined by the molecular set up of the inhibitory agent [64]. The development of novel agents with increased half-life of the drug-target complex, such as panobinostat, could well be a promising strategy. On the clinical side, studies involving HDACis should demonstrate on target activity in tumor tissue in addition to simply monitoring drug levels and histone acetylation in PBMCs as a surrogate), and possibly develop novel dosing and/or application schedules, such as oral vs. intravenous application, extended release or infusion over several hours, multiple dosing with lower doses etc.

## Conclusions

In summary, we conclude, that i) class I HDACs in general and HDAC2 in particular are a valid target in Group 3 MB, ii) *MYC* amplified MB are more sensitive to HDACi than *MYC* non-amplified MB and thus *MYC* amplification could serve as a positive predictive marker for HDACi treatment, and iii) that both inhibitory profiles and binding kinetics of compounds are of major importance when designing clinical trials using HDACis.

## Additional files

Additional file 1: Table S1. patient characteristics. Additional file 2: Table S2. siRNAs used in this study. Additional file 3: Table S3. PCR primers used in this study. Additional file 4: Table S4. Calculated EC50s.
